# Effect of Portal Glucose Sensing on Systemic Glucose Levels in SD and ZDF Rats

**DOI:** 10.1371/journal.pone.0165592

**Published:** 2016-11-02

**Authors:** Atanu Pal, David B. Rhoads, Ali Tavakkoli

**Affiliations:** 1 Department of Surgery, Brigham and Women’s Hospital, 75 Francis Street, Boston, MA, 02115, United States of America; 2 Harvard Medical School, 25 Shattuck Street, Boston, MA, 02115, United States of America; 3 Pediatric Endocrine Unit, MassGeneral Hospital *for* Children, 55 Fruit Street, Boston, MA, 02114, United States of America; 4 Center for Weight Management and Metabolic Surgery, Brigham and Women’s Hospital, 75 Francis Street, Boston, MA, 02115, United States of America; Max Delbruck Centrum fur Molekulare Medizin Berlin Buch, GERMANY

## Abstract

**Background:**

The global epidemic of Type-2-Diabetes (T2D) highlights the need for novel therapeutic targets and agents. Roux-en-Y-Gastric-Bypass (RYGB) is the most effective treatment. Studies investigating the mechanisms of RYGB suggest a role for post-operative changes in portal glucose levels. We investigate the impact of stimulating portal glucose sensors on systemic glucose levels in health and T2D, and evaluated the role of sodium-glucose-cotransporter-3 (SGLT3) as the possible sensor.

**Methods:**

Systemic glucose and hormone responses to portal stimulation were measured. In Sprague-Dawley (SD) rats, post-prandial state was simulated by infusing glucose into the portal vein. The SGLT3 agonist, alpha-methyl-glucopyranoside (αMG), was then added to further stimulate the portal sensor. To elucidate the neural pathway, vagotomy or portal denervation was followed by αMG+glucose co-infusion. The therapeutic potential of portal glucose sensor stimulation was investigated by αMG-only infusion (vs. saline) in SD and Zucker-Diabetic-Fatty (ZDF) rats. Hepatic mRNA expression was also measured.

**Results:**

αMG+glucose co-infusion reduced peak systemic glucose (vs. glucose alone), and lowered hepatic G6Pase expression. Portal denervation, but not vagotomy, abolished this effect. αMG-only infusion lowered systemic glucose levels. This glucose-lowering effect was more pronounced in ZDF rats, where portal αMG infusion increased insulin, C-peptide and GIP levels compared to saline infusions.

**Conclusions:**

The portal vein is capable of sensing its glucose levels, and responds by altering hepatic glucose handling. The enhanced effect in T2D, mediated through increased GIP and insulin, highlights a therapeutic target that could be amenable to pharmacological modulation or minimally-invasive surgery.

## Introduction

The epidemic of Type 2 Diabetes (T2D) affects almost 400 million people worldwide [1] and is predicted to increase in parallel with the obesity epidemic. This calls for safe and cost-effective treatments that can be made available globally. Current anti-diabetic drugs have limited efficacy, with less than 50% of patients achieving target HbA1c [2]. Bariatric surgery is the most successful anti-diabetic intervention, but many patients do not qualify for this surgery or desire the intervention, which highlights the need for novel therapeutic targets with improved efficacy. Studies into the anti-diabetic effects of bariatric surgery have identified changes in post-operative intestinal glucose handling and subsequent alterations in portal glucose levels as a possible mechanism [3] (also Pal et al 2016 under review).

There has been much recent interest in portal glucose sensing [4]. It is well established that pancreatic β-cells have a major role in glucose homeostasis by sensing *systemic* glucose levels [5]. However, absorbed nutrients first enter circulation through the portal vein, so portal nutrient sensing may therefore be critical in anticipating a nutrient load and preparing the liver for it. Indeed, the notion of a portal glucose sensor regulating systemic function is supported by studies showing that portal glucose infusion can lead to systemic hypoglycemia [6] and reduced food intake [7] in rodents and increased hepatic glucose uptake in the dog [8].

However, several issues remain unresolved.

1. The molecular identity of the portal glucose sensor is unclear with both the facilitated glucose transporter-2 (GLUT2) [9] and the sodium-glucose cotransporter-3 (SGLT3) [7, 10] proposed as candidates.
2. Portal glucose sensing requires further study in the post-prandial phase.
3. Changes in portal glucose sensing in the pathophysiology of T2D have not been studied.

We hypothesize that portal glucose sensing has a significant physiological role in the post-prandial phase and that this pathway may be a therapeutic target in the diabetic state.

We therefore aim to characterize: (i) the molecular and neural elements of portal glucose sensing, (ii) its downstream hormone and hepatic effects, and (iii) the effect of a diabetic phenotype on this sensing mechanism. Based on earlier studies on intestinal nutrient sensing [11], we focused the current experiments on the role of SGLT3 as the relevant glucose sensor.

## Methods

### Animals and catheter placement

Animal studies were performed in accordance with protocols prospectively approved by the named Institutional Animal Care and Use Committee (IACUC) and this specific study was approved by the Harvard Medical Area Standing Committee on Animals. Male Sprague-Dawley (SD; 220–240g, Harlan, IN) and male Zucker Diabetic Fatty (ZDF, 13 weeks, Charles River) rats were used. The SD rat is a healthy rodent model. The ZDF rat is an obese diabetic model, which has a mutation in the leptin receptor gene (*fa* gene) leading to polyphagia, obesity, glucose intolerance and fasting hyperglycemia [12]. We selected 13-week old ZDF rats with fasting glucose >300mg/dl to reduce the variability in the diabetic phenotype.

Rats were acclimated for 7 days under a 12:12 light: dark cycle (lights-on 7 AM) with *ad libitum* access to standard rat chow (Purina 5053) for SD rats and high-fat diet (Purina 5008) for ZDF rats. After an overnight fast, they were anesthetized using isoflurane (1–3% in oxygen). Experiments were consistently started at 9 AM to avoid the confounding factor of diurnal variation and conducted under anesthesia to allow for accurate catheter placement and organ harvest at the end of the experiment. At the endpoint of the experiment, animals were sacrificed under general anesthesia by exsanguination followed by bilateral thoracotomy.

During the portal infusion over 90 min, systemic venous blood was sampled before (0 min) and at 10, 30, 60 and 90 min after the start of the infusion (S1 Fig). For systemic sampling, a silastic catheter (Dow Corning, ID 0.02in) was advanced through the left jugular vein into the right atrium, allowing sampling of mixed systemic venous blood. For portal infusion, a silastic catheter (Dow Corning, ID 0.012in) was inserted into the superior mesenteric vein (SMV). The catheter was only advanced 1cm to allow mixing of the infusate with portal blood.

Several portal infusion groups were used, with infusions of glucose, αMG, or in combination, with or without denervation procedures (S1A and S1B Fig). Animals were kept warm throughout the experiment to avoid the effects of changing body temperature on glucose metabolism, and respiratory rate kept constant to ensure constant depth of anesthesia.

### Agonist-glucose dual infusion experiments: SD rats

To determine the role of portal SGLT3 on glucose tolerance, αMG was co-infused with glucose (αMG+Glu; n = 5) in SD rats, and systemic venous blood sampled. αMG (Sigma-Aldrich, St Louis, MO) was selected as the agonist because it binds SGLT3 to cause membrane depolarization [13]. Glucose (1g/kg = 5.5mmol/kg) and αMG (1.08g/kg = 5.5mmol/kg) was dissolved in 7.2ml/kg water and infused over 90 min (0-90min; i.e. αMG 60 μmol kg^-1^ min^-1^ + glucose 60 μmol kg^-1^ min^-1^). The infusion volume was limited to approximately 10% of the blood volume (estimated at 70ml/kg) to minimize circulatory perturbations. For example, a 250g rat would receive 0.25g glucose and 0.27g αMG (dissolved in total volume of 1.8ml water, infused at 20 μl per min for 90 min). The control group for these dual infusions received an isomolar saline-glucose (Sal+Glu; n = 5; 5.5mmol/kg glucose and 5.5mmol/kg saline) infusion. Glucose (5.5mmol/kg) and 3-O-methyl-glucose (3-OMG; 5.5mmol/kg) co-infusion studies were also performed to investigate the role of SGLT1 and GLUT2.

### Surgical denervation models

To elucidate neural elements of the portal sensing pathway, SD rats were subjected to either truncal vagotomy, or portal denervation, or sham portal denervation. In the truncal vagotomy group (VAG, n = 5), a sub-diaphragmatic vagotomy was performed 30 min before the portal αMG-glucose co-infusion. For the vagotomy, the anterior and posterior vagal trunks were identified under an operating microscope and divided at the sub-diaphragmatic level as previously described [14].

In the portal denervation group, the portal vein was denervated prior to portal αMG-glucose co-infusion (CAP, n = 6). For portal denervation, the portal vein was treated with capsaicin, which behaves as a neurotoxin in the dose used leading to deafferentation of nerves [15] and has been previously used for portal denervation [7]. The portal vein was visualized by retracting the liver cephalad and the intraperitoneal part of the duodenum to the left. A piece of sterile gauze was gently passed through the epiploic foramen and around the portal vein (S1C Fig). Sterilized Parafilm was positioned around the gauze to prevent exposure of other tissues to capsaicin. A solution of capsaicin (1 mg; Sigma-Aldrich, St Louis, MO) in 1ml of vehicle (10 μl ethyl alcohol, and made up to 1ml with 90% olive oil and 10% Tween-80; Sigma-Aldrich) was applied to the gauze for 30 min as previously described [16]. The gauze and Parafilm were then removed. Rats were allowed to recover for two weeks before performing portal infusion experiments. For sham portal denervation (SHAM, n = 5), the portal vein was treated with vehicle only.

### Agonist-only single infusion experiments: SD and ZDF rats

To determine whether stimulation of portal SGLT3 (without glucose) had a glucose-lowering effect, αMG 2.16g/kg (11mmol/kg) dissolved in 7.2ml/kg water was infused into the portal veins of SD rats over 90 min (n = 5; SD- αMG; αMG 120μmol kg^-1^ min^-1^), with the control group receiving an isomolar saline infusion (SD-Sal, n = 5; 11mmol/kg). To investigate whether the portal sensing pathway was altered in T2D, these agonist-only experiments were also performed in ZDF rats (ZDF- αMG, n = 4, and ZDF-Sal, n = 4).

At the end of each experiment, the liver was harvested, snap-frozen in liquid N_2_, and stored at -80°C for later mRNA expression analyses.

### Blood analysis

Systemic blood glucose level was measured at each time-point using a glucometer (LifeScan OneTouch, Milpitas, CA). For hormone analysis, blood was centrifuged at 4°C 4000*g* for 15 min, the plasma aspirated, and stored at -80°C. Systemic plasma GIP, GLP-1, glucagon, insulin and C-peptide levels were measured at the 0 and 90 min time points in duplicate using the Milliplex rat metabolic hormone panel (catalog number RMHMAG-84K, Millipore, Darmstadt, Germany).

### Real-time PCR for mRNA expression

The hepatic mRNA expression of several enzymes and proteins involved in glucose metabolism was measured relative to β-actin (internal reference). They were glucokinase, enzymes of gluconeogenesis (glucose-6-phosphatase, G6Pase; and phosphoenolpyruvate carboxykinase, PEPCK), enzymes of glycogen metabolism (glycogen synthase, GS; glycogen phosphorylase, GP) and the carbohydrate responsive element binding protein (ChREBP). Primer sequences are shown (Table 1).

**Table 1:** 

	Forward primer	Reverse primer
Gluco-kinase	5’-GTGGTGCTTTTGAGACCCGTT-3’	5’-TTCGATGAAGGTGATTTCGCA-3’
PEPCK	5’-CTCACCTCTGGCCAAGATTGGTA-3’	5’-GTTGCAGGCCCAGTTGTTGA-3’
G6Pase	5’-AACGTCTGTCTGTCCCGGATCTAC-3’	5’-ACCTCTGGAGGCTGGCATTG-3’
ChREBP	5’-GGGACATGTTTGATGACTATGTC-3’	5’-AATAAAGGTCGGATGAGGATGCT-3’
GS	5’-CTGCATGGGAAGCTGAAAGACTCCCCG-3’	5’-CATGGCTCTACTTGAGTCTTCACATTA-3’
GP	5’-CCAAAGATCCAGACTGCTTCAAGGATG-3’	5’-CCAGAGCAGGCTATATTTCTGATCACCTTT-3’
β-actin	5’-GGAGATTACTGCCCTGGCTCCTA-3’	5’-GACTCATCGTACTCCTGCTTGCTG-3’

RNA was extracted from tissue samples using the mirVana mRNA Isolation Kit (Ambion) and quantified (Spectramax M5; Molecular Devices). 2 μg of RNA was reverse-transcribed (Superscript III and oligo-dT, Invitrogen) to form cDNA. Real-time PCR was then performed (Applied Biosystems ABI 7900HT) on a 384-well plate using SYBR Green (Life Technologies).

### Calculations

**Fig 1:**
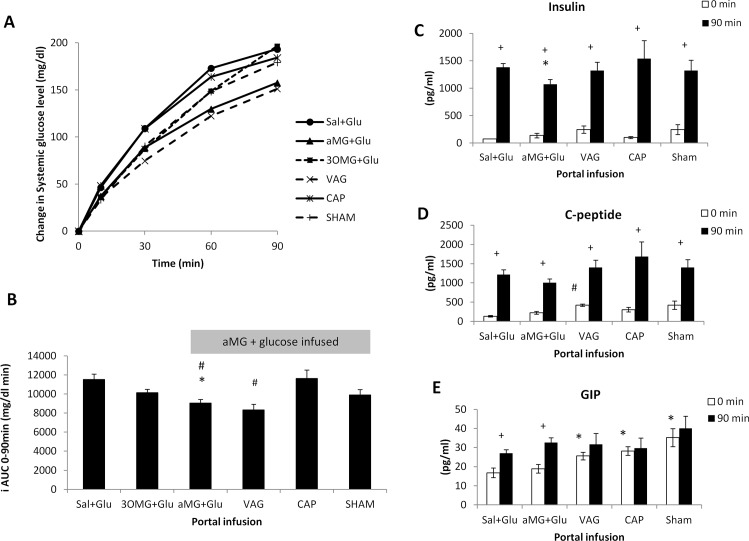
Systemic glucose levels and hormone levels during portal agonist-glucose dual infusion experiments. (A) Systemic glucose levels over the course of the portal infusion. (B) Incremental AUC (N = 5–6 per group). Hormone levels at 0 min (before the start of the portal infusion) and 90 min (at the end of the portal infusion), compared across the different infusion groups. (C) Insulin levels. (D) C-peptide levels. (E) GIP levels. * p<0.01 vs. Sal-Glu; # p<0.05 vs. CAP; + p<0.01 vs. 0 min.

To assess the effects of the portal infusions on systemic glucose level, change in systemic glucose from 0 to 90 min, and the incremental area-under-curve (iAUC) of systemic glucose level was calculated (Fig 1D and 1E).

### Statistical analysis

Data analysis was performed using Excel. The two-tailed unpaired t-test was used for planned comparison of two groups. For comparing several treatment groups to a single control group, Analysis of variance (ANOVA) with post-hoc analysis (Tukey-Kramer method) was used. Data are presented as mean +/- standard error of the mean.

## Results

Pilot experiments showed that portal glucose infusion caused a rise in systemic glucose level, rather than the hypoglycemia demonstrated by Burcelin et al in a mouse model (discussed below). The agonist-glucose dual infusion experiments were therefore designed to determine whether the addition of αMG, which binds to SGLT3 to cause membrane depolarization and receptor stimulation, affected the rise in systemic glucose level. Raw data is included (S1 Data).

### Dual αMG and glucose infusions in SD rats

#### Systemic blood glucose

Co-infusion of αMG+Glu lowered peak glucose level ~10% compared with Sal+Glu (systemic glucose level at 90 min 264 vs. 299mg/dl, p<0.05, Fig 1A) and lowered iAUC ~20% (9,036 vs. 11,517 mg/dl min, p<0.01, Fig 1B). In contrast, co-infusion of 3OMG+Glu (more specific SGLT1 agonist) did not have a significant effect (iAUC 10,138mg/dl min, NS, Fig 1B).

The effect of αMG was unaffected by vagotomy and so peak glucose and iAUC were still lower than Sal+Glu (254mg/dl and 8,341mg/dl min; p = 0.01 and p<0.01, respectively). Portal denervation by capsaicin worsened glucose levels and abolished the effect of αMG (peak glucose 286mg/dl; iAUC 11,449mg/dl min, p<0.05 vs. αMG+Glu, Fig 1B). Sham portal denervation had no effect (iAUC 9,901 mg/dl min; p<0.05 vs. Sal+Glu).

#### Hormone

There were no differences in baseline insulin levels (0 min) among the four SD rat groups in these experimental arms. In all groups, the insulin level at 90 min was greater than that at 0 min (p<0.01, Fig 1C), consistent with the rise in systemic glucose level. Like insulin, C-peptide levels at 90 min were greater than at 0 min in all groups (p<0.01, Fig 1D). Insulin level at 90 min was lower in αMG-Glu than in Sal-Glu (p<0.05), consistent with the lower systemic glucose levels in this group.

Systemic GIP levels were increased at 90-min in both portal Sal+Glu and αMG+Glu groups (‘porto-incretin reflex’ discussed below); GIP levels were however similar between the two groups (27.0 vs. 32.6 pg/ml at 90 minutes; p = 0.13; Fig 1E). Following vagotomy, capsaicin, and also sham denervation, baseline GIP levels were increased compared to animals with no portal manipulation (Fig 1E), but subsequent portal infusions did not lead to a further increase in GIP levels (discussed below). There were no changes in GLP-1 or glucagon level during the portal infusions in any of the groups (0 vs. 90 min, NS), with levels in all groups <60pg/ml and <20pg/ml, respectively (data not shown).

#### Hepatic mRNA expression

**Fig 2:**
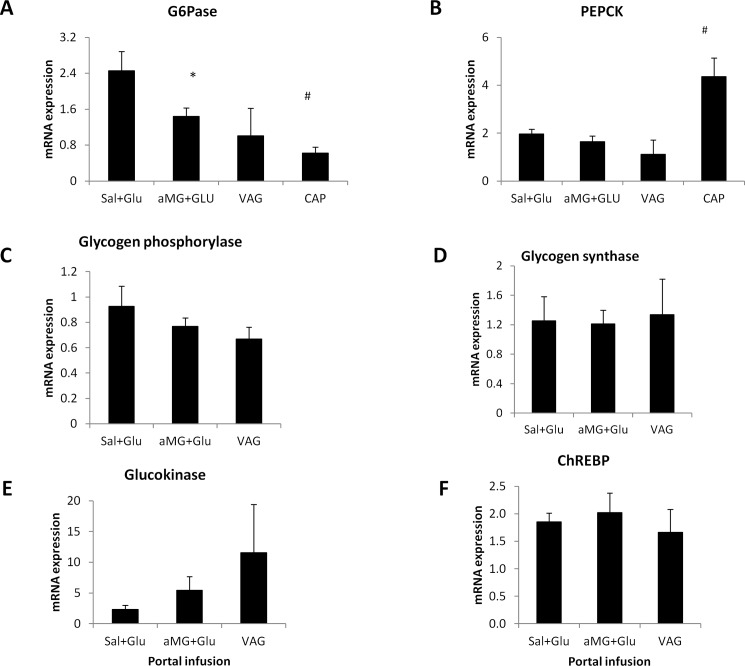
mRNA expression during portal agonist-glucose dual infusions. mRNA expression of Sal+Glu, αMG+Glu, VAG and CAP groups: (A) glucose-6-phosphatase, (B) PEPCK, (C) glycogen phosphorylase, (D) glycogen synthase (E) glucokinase, and (F) ChREBP. * p<0.05 vs. Sal-Glu.

In SD rats, αMG+Glu co-infusion reduced G6Pase expression (p<0.05) and this decrease was maintained despite vagotomy. There was a non-significant increase in glucokinase expression in αMG+Glu (p = 0.23) and VAG (Fig 2). There were no significant changes observed in expression of ChREBP, GP or GS. Of note, in the CAP group there was a significant decrease in G6Pase expression and a significant increase in PEPCK expression (Fig 2) (discussed below).

### αMG-only single infusions in SD and ZDF rats

To determine whether stimulating portal SGLT3 alone (i.e. without glucose) would cause a decrease in systemic glucose level and therefore be of potential therapeutic value, αMG was infused alone and compared to saline infusions as control.

#### Systemic blood glucose

**Fig 3:**
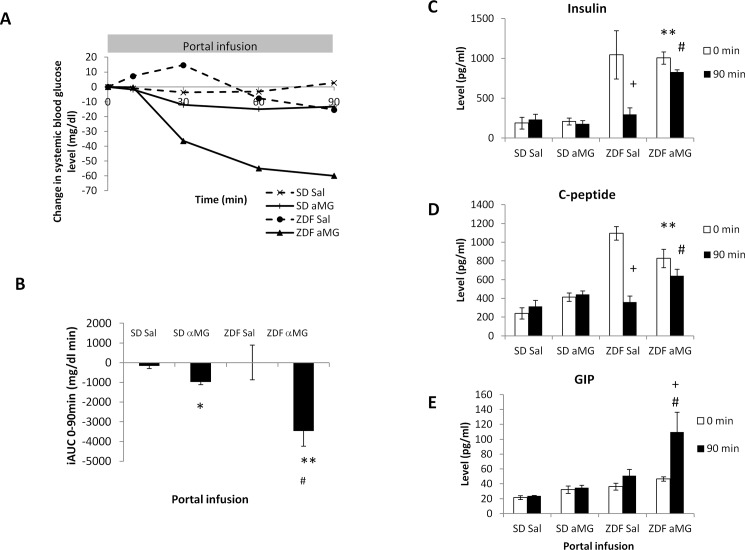
Systemic glucose levels and hormone levels during portal agonist-only single infusion experiments. Effect of portal αMG infusion on (A) change in systemic glucose levels in the fasting state and (B) incremental AUC (N = 4–5 per group). Hormone levels at 0 min (baseline) and 90 min (at end of portal infusion), compared across groups. (C) Insulin levels. (D) C-peptide levels. (E) GIP levels. *p<0.01 vs. SD Sal; **p<0.01 vs. SD αMG; # p<0.05 vs. ZDF Sal, + p<0.01 vs. 0 min.

In SD rats, portal αMG infusion led to a decrease in systemic glucose level compared with saline infusion (change in systemic glucose level at 90 min -13.4 vs. +2.8mg/dl, p<0.05; iAUC -983 vs. -165mg/dl min, p<0.01; Fig 3A and 3B). In ZDF rats, as expected the baseline glucose level was higher (ZDF vs. SD rats 451 vs. 104mg/dl). Portal αMG also lowered systemic glucose level compared with saline infusion (change in glucose level -60.0 vs. -15.5mg/dl, p<0.05; iAUC -3,466 vs. +125mg/dl min, p<0.05). The absolute effect size of αMG was much more pronounced in ZDF rats than in SD rats (change in glucose level -60.0 vs. -13.4mg/dl, p<0.05; iAUC -3,466 vs. -983mg/dl min, p<0.01); the relative effect size was similar (-13.3% vs. -12.9%).

#### Hormone

Baseline insulin and C-peptide levels were higher in ZDF than in SD rats (e.g. insulin SD- αMG vs. ZDF- αMG, 209 vs. 1005pg/ml, p<0.0001; Fig 3C and 3D), which is consistent with the insulin resistant phenotype of the ZDF rat. There were no differences in baseline GIP levels (Fig 3E).

In SD rats, saline and αMG infusions did not affect the levels of insulin, C-peptide or GIP at 90-minutes, likely due to only modest changes in systemic glucose levels (Fig 3).

In ZDF-Sal rats, insulin and C-peptide levels decreased at 90 min compared with 0 min (p = 0.05 and p<0.001, respectively). In contrast, insulin and C-peptide levels remained high throughout the infusion in ZDF- αMG rats and were thus higher at 90 min than in ZDF-Sal rats (p<0.05). A further observation in the ZDF- αMG group was that GIP levels at 90 min were higher than at 0 min (47 vs. 108pg/ml, p<0.05; Fig 3E). This effect was not seen in ZDF-Sal. This result suggests that portal αMG infusion increased GIP levels and prevented the drop in insulin secretion observed in ZDF-Sal rats (‘porto-incretin reflex’ discussed below).

There were no changes in GLP-1 or glucagon level during the portal infusions in any of the groups (0 vs. 90 min, NS), with levels in all groups <60pg/ml and <20pg/ml, respectively (data not shown).

#### Hepatic mRNA expression

**Fig 4:**
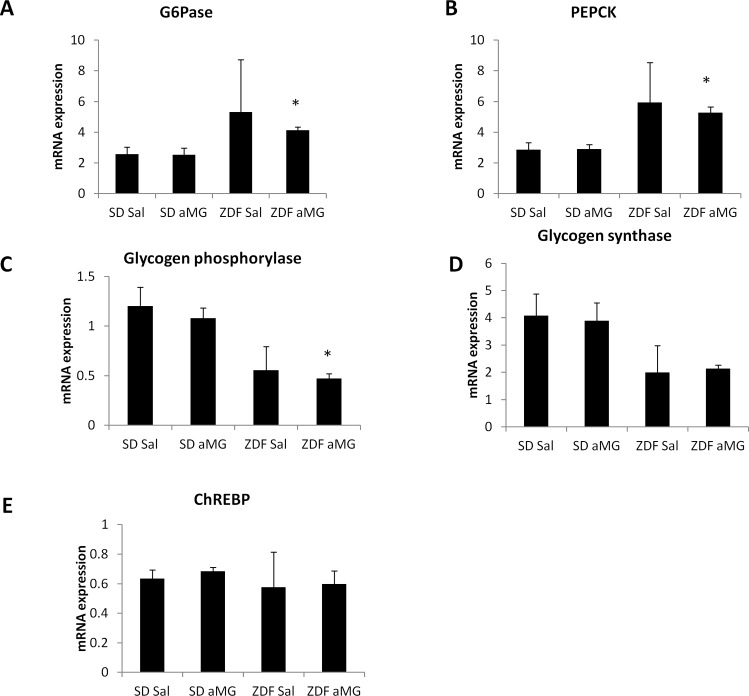
mRNA expression during portal agonist-only single infusions. mRNA expression of SD-Sal, SD- αMG, ZDF-Sal and ZDF-αMG: (A) glucose-6-phosphatase, (B) PEPCK, (C) glycogen phosphorylase, (D) glycogen synthase, and (E) ChREBP. * p<0.05 vs. SD αMG.

There were some differences in expression between the two strains. Expression of the genes involved in gluconeogenesis (PEPCK, G6Pase) were higher in ZDF rats (p<0.05), and expression of those involved in glycogen metabolism (GS, GP) were lower (p = 0.09 and p<0.05, respectively). Within the strains, there were no differences in expression of any of these genes after saline or αMG infusions (Fig 4).

## Conclusions

In this study we demonstrate that (i) glucose sensing, likely by portal SGLT3, improves tolerance to a portal venous glucose load by a pathway that is neurally, but not vagally, mediated, (ii) the absolute effect size of this pathway is enhanced in a diabetic rodent model, and (iii) portal sensing has downstream effects on hormone levels and hepatic gene expression. These results highlight the therapeutic potential of portal glucose sensors and SGLT3 in T2D.

The concept of portal glucose sensing arose from several studies that showed glucose delivered into the portal vein mediates effects that do not occur if an equivalent amount of glucose is delivered directly into the systemic circulation [17]. In canines, portal glucose infusion increased hepatic glucose uptake, which was abolished by denervating the liver and portal vein [18]. In pigs, portal glucose infusion also caused marked systemic hyperglycemia [19]. While these systemic glucose increases were not unexpected, a similar study in mice elicited the opposite effect [6], where portal glucose infusion caused a seemingly paradoxical systemic hypoglycemia. Such data have generated interest in characterizing the portal glucose sensor, with particular focus on SGLT3 and GLUT2. SGLT3 is expressed in the portal vein and portal infusion of its agonist, αMG, reduced food intake in the rat [6]. In a knockout model, GLUT2 has been implicated in the glucose sensing pathway [9].

Our study focused on the role of SGLT3 and we demonstrate for the first time, to our knowledge, the glucose lowering effect of targeted stimulation of the portal *sensor* and therefore its therapeutic potential. The infusion rate in our study was selected to replicate a post-prandial glucose load (Pal et al 2015). In the αMG+Glu group the rise in systemic glucose was less marked than in the control group, suggesting that additional stimulation of SGLT3 by αMG has a glucose-lowering effect. Elimination of this effect by portal denervation but not vagotomy indicates signaling from the portal vein through a non-vagal neural pathway, and therefore through the dorsal spinal afferent pathway [20]. This is consistent with work by others which showed the effects of portal glucose sensing on food intake were abolished by portal denervation [7], and that vagal blockade by cooling did not affect net hepatic glucose uptake [21].

To elucidate the basis for this glucose-lowering effect, we measured systemic hormone levels and hepatic gene expression. The decrease in hepatic G6Pase expression implies that an increase in net hepatic glucose uptake is the primary effect of portal sensing in the SD rat, and consistent with previous reports of increased hepatic glucose uptake following portal glucose infusions [8, 17]. We do need to confirm changes in protein level of these enzymes to further validate this hypothesis as it is possible that 90-minutes would be insufficient time to substantially change protein levels. This effect would be an interesting avenue for future research. A surprising finding in the CAP group was the decreased G6Pase expression yet increased PEPCK expression. It may be that the chronically impaired function of the portal sensor in this group led to increased PEPCK expression, and that reduced G6Pase expression may be a compensatory mechanism.

To help identify the portal glucose sensor we used αMG infusions to assess a role for SGLT3. We acknowledge that αMG is an agonist of both SGLT1 and SGLT3. However to rule out SGLT1-mediated effects, we also performed co-infusion studies using glucose and the SGLT1-specific agonist 3-OMG which had no effect on systemic glucose levels. Furthermore, SGLT1 expression has not been specifically reported in the portal vein. Importantly, 3-O-methyl-glucose can also stimulate GLUT2, and absence of a response in the above infusion studies provides further support that GLUT2 is unlikely to be involved. SGLT3 is expressed elsewhere in the body including skeletal muscle [9] which raises the possibility that portal αMG administration may have its affect by eventually reaching an extra-hepatic site; however, the portal denervation group in our study helps isolate the effects of glucose sensing at the portal locus.

We also identify a novel role for portal sensing in the regulation of GIP secretion. Glucose infusion in the SD rat (in both the αMG+Glu and Sal+Glu groups) increased 90 min systemic GIP levels compared to 0 min. This effect was abolished by portal denervation and by vagotomy. This suggests that portal glucose sensing (via portal innervation) stimulates intestinal GIP secretion (via the vagus nerve) in what we have called the ‘porto-incretin reflex’. The efferent limb of the pathway we suggest is consistent with the anatomical distribution of the vagus nerve to the small intestine [19]. Human studies have suggested a role for the vagus nerve in GIP secretion as the GIP response was decreased following vagotomy [22]. GIP levels did not differ between αMG+Glu and Sal+Glu groups so we do not believe this can explain the difference in glucose levels in the SD rat. However, the porto-incretin reflex may lead to increased insulin levels in the ZDF- αMG group and underpin the therapeutic benefit of portal SGLT3 in T2D (discussed below). Baseline GIP levels were also elevated in the portal denervation sham group and suggests that portal manipulation alone can lead to the changes in GIP levels, and this is mediated through pathways that are not capsaicin specific.

Our *agonist-only experiments* showed that stimulation of SGLT3 alone, without the presence of glucose, lowered systemic glucose level by 13 mg/dl in the SD rat. The absolute effect size was over 4 times larger in the ZDF rat, with αMG causing a decrease of 60 mg/dl. Although the relative effect size was similar (∼13%), we highlight the critical role of SGLT3 in mediating an enhanced glucose-lowering effect in a diabetic phenotype, without inducing hypoglycemia in normoglycemic animals. Both of these properties are useful when considering portal SGLT3 as a therapeutic target, with the effect size increasing with degree of hyperglycemia.

In the ZDF rat, the glucose-lowering effect of αMG is likely hormone-mediated. The 90 min GIP, insulin and C-peptide levels were higher in ZDF- αMG than ZDF-Sal. We therefore propose that stimulation of portal SGLT3 increases GIP secretion through an exaggerated ‘porto-incretin reflex’ described above, which maintains insulin secretion throughout the 90 min portal infusion, leading to the observed decrease in glucose level. This is in contrast to the ZDF-Sal group where, without SGLT3 stimulation and GIP secretion, the 90 min insulin level dropped markedly. The reason for this drop in insulin level is unclear. Although isoflurane anesthesia has been shown to reduce insulin secretion [23], we believe this is unlikely as this was not seen in our other experimental groups. It is also noteworthy that this neural mechanism continues to be effective despite the use of isoflurane anesthesia. Isoflurane has been shown to inhibit medullary 5-HT neurons [24], but our data shows that portal sensing remains intact.

The rise in GIP levels was not seen in the SD- αMG group, suggesting that the ‘porto-incretin reflex’ is upregulated in diabetes and may reflect a pathophysiological regulatory mechanism. Furthermore it has previously been shown that insulin is required to increase hepatic glucose uptake in response to portal glucose delivery [25]. The high insulin levels in ZDF- αMG rats may act through this mechanism to reduce systemic glucose level. We conclude that in SD rats the reflex is set such that it does not allow glucose to drop into the hypoglycaemic range. This observation and hypothesis warrants further investigation. Our experiments did not identify a role for GLP-1 and suggest that GLP-1 secretion requires the presence of intestinal luminal glucose.

The patterns of hepatic mRNA expression following single infusions did not add further mechanistic insights. There may be changes in hepatic glucose fluxes in response to portal glucose sensing, but studies using labeled glucose would be required to measure this. A limitation of our study is that we did not measure protein levels and enzyme activity, which would further elucidate hepatic changes.

Previous studies have shown that portal glucose sensing by SGLT3 in the post-absorptive phase reduces future food intake i.e. the next meal [7], and portal glucose infusion does not terminate the current meal [26]. The source of glucose for this post-absorptive effect is thought to be intestinal gluconeogenesis. In this study we show, for the first time, that portal sensing by SGLT3 has a role in the post-prandial phase to improve glucose tolerance to an ongoing meal, an effect that may have important therapeutic implications.

In summary, our data shows that (i) portal glucose sensing, likely by SGLT3, improves tolerance to a portal venous glucose load by a neural non-vagal pathway; (ii) the diabetic phenotype is associated with an augmented glucose-lowering effect of the portal sensor, which may be mediated through an exaggerated ‘porto-incretin reflex’ by increased GIP secretion to maintain high insulin levels. Our study has particular therapeutic relevance because we identify a pathway that continues to have a regulatory effect even in the diabetic state and is therefore a potential therapeutic target. This raises the prospect of modulation of this pathway through minimally invasive or pharmacological means that target portal innervation.

## Supporting Information

S1 Data Minimal data set. Raw data for glucose levels, hormone levels and mRNA levels for each experimental group.
(XLSX)

S1 Fig Experimental design and systemic blood glucose changes. Timeline of experiment: (A) agonist-glucose dual infusion and (B) agonist-only single infusion experiments, and systemic blood sampling schedule. (C) Portal denervation surgery. Typical curves showing (D) systemic glucose level during agonist-glucose dual infusion experiment, and (E) change in systemic glucose during agonist-only single infusion experiment, with incremental area-under-curve (iAUC) shaded.
(TIF)
