# Supplementary figures and images for: Effect of Portal Glucose Sensing on Systemic Glucose Levels in SD and ZDF Rats
Source: PLoS One. 2016 Nov 2;11(11):e0165592. doi: 10.1371/journal.pone.0165592 (PMC5091783; doi:10.1371/journal.pone.0165592)

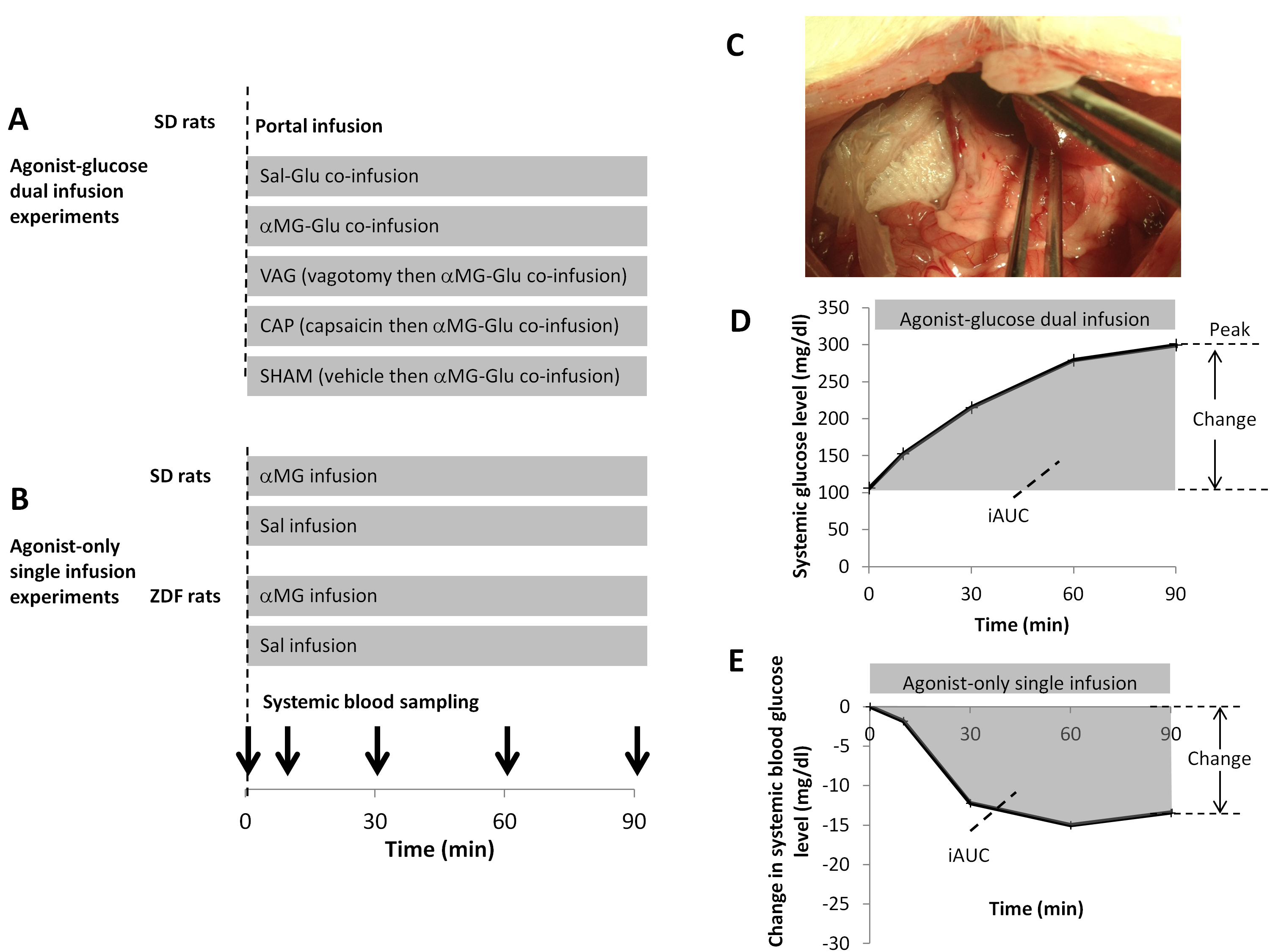

Supplement: S1 Fig — Timeline of experiment: (A) agonist-glucose dual infusion and (B) agonist-only single infusion experiments, and systemic blood sampling schedule. (C) Portal denervation surgery. Typical curves showing (D) systemic glucose level during agonist-glucose dual infusion experiment, and (E) change in systemic glucose during agonist-only single infusion experiment, with incremental area-under-curve (iAUC) shaded. (TIF) [file pone.0165592.s002.tif]
